# Boredom in the COVID-19 Pandemic

**DOI:** 10.3390/bs12110428

**Published:** 2022-11-01

**Authors:** James Danckert

**Affiliations:** Department of Psychology, University of Waterloo, 200 University Avenue West, Waterloo, ON N2L 3G1, Canada; jdancker@uwaterloo.ca

## 1. Introduction

The past two and half years have been witness to an extraordinary global pandemic with obvious and devastating health outcomes. At the time of writing, despite many governments lifting any restrictions initially intended to curb the spread of COVID-19, the pandemic is not over. By some estimates, we are in the midst of the eighth wave, with hospitalizations and deaths once again rising. However, it is the earlier phase of the pandemic upon which this Special Issue is focused, a time when many governments imposed restrictions on movement and social gatherings. From the very first imposition of so-called ‘lockdowns’, it was evident that, as a society, we were bracing for a widespread challenge to everyday existence, one we had not anticipated—boredom. Indeed, across 116 countries, there was a slight increase in reported feelings of boredom [1]. Boredom was certainly not the only challenge to mental health on the horizon, and data have since confirmed that mental health indeed suffered under the constraints imposed by the extraordinary circumstances [2]. However, the potential for a society-wide increase in felt boredom offered researchers a unique opportunity to study the phenomenon ‘in-the-wild’. This Special Issue brings together a broad range of papers relevant to both in-the-moment feelings of boredom (i.e., state boredom) and the individual propensity to experience the state more intensely (i.e., trait boredom proneness). Not only are there insights to be gained for our understanding of this ubiquitous human experience, but there is also potential utility for public policy if we can heed what boredom in the pandemic is telling us.

During the earliest stages of the pandemic (March/April 2020), in which restrictions were perhaps the most ubiquitous, at least in Western cultures, work showed that those more prone to boredom were also more likely to break the rules of social distancing [3,4]. This accords with theoretical accounts of state boredom as a sign of rising opportunity costs [5,6]. Indeed, the imposition of constraints to movement that led many to work from home and, where possible, to avoid gatherings (e.g., by shopping online, even for basics such as groceries) made starkly evident the loss of typical outlets for action. In other words, every day highlighted the high level of opportunity costs, given that we were prevented from engaging with life in our usual ways. It is worth pointing out that the in-the-moment feelings of boredom themselves were not responsible for increased rule-breaking behaviours [1]. However, for the highly boredom prone, those who feel boredom more frequently and intensely [7], rising opportunity costs made it more difficult to adhere to the strictures of lockdowns. One might have hoped that, with more experience of these restrictions, the influence of trait boredom would wane, perhaps as a function of improved coping skills. Unfortunately, this was not the case [8]; but see [9] for work showing that planning for boredom improved outcomes]. Derived from data obtained one year into the pandemic, Drody and colleagues replicated the relationship between boredom proneness and rule breaking. This highlights the challenge faced by those of high boredom proneness in dealing with constraint. For policymakers, it may be important to emphasise the social contract inherent to impositions or to simultaneously encourage alternative outlets for engagement [9].

It is worth pointing out that it is not the case that choices made by the boredom prone are driven by antisocial desires to break rules—instead, they are likely borne of the desire to eliminate the feelings of boredom. In at least one study, this was coupled with the desire to engage in prosocial behaviours [10]. Regardless, it is feasible to promote more adaptive outlets for coping with pandemic boredom. Brosowsky and colleagues [11] showed that those who engaged in more creative outlets during the pandemic tended to have better mental health outcomes. This was not necessarily about grand creative endeavours—large-scale canvasses, sculptures, or operettas—but, rather, everyday activities that gave one a sense of agency and accomplishment. Unfortunately, the highly boredom prone were less likely to engage in such activities [11]. Clearly, addressing the challenges of coping with boredom in positive ways is critical in managing the kind of exceptional circumstances brought on by the pandemic (also see [12,13]). Indeed, a focus on positive psychology more generally has been shown to lead to better outcomes for coping in the pandemic [14,15].

The challenge of boredom under such obvious constraints may seem plainly evident —how does one entertain oneself? While there is debate as to whether the construct of boredom proneness is itself singular [16], work by Van Dang and Lench [17] showed, through serial mediation, that the need for internal stimulation (or, more colloquially, the need or capacity to entertain oneself) led to an increased intensity of feelings of boredom via an elevated frequency and duration of boredom episodes. It will be important to replicate this model in a sample free from the constraints of the pandemic to determine the robustness of the mediation under less-restricted times. What it suggests is that, for boredom, the intensity, frequency, and duration of individual episodes interact, potentially in multiplicative ways. Interestingly, there are potential real-world outcomes (beyond merely rule breaking) for this struggle to entertain oneself. Van Tilburg and colleagues [18] showed that ratings of boredom during the pandemic were associated with increased weight gain. Interestingly, in their sample, the eating behaviour was driven by convenience as opposed to health-related choices. In other words, eating was a time filler when other outlets for action were absent. People did not report that their food choices were motivated by either novelty seeking or the desire to regulate their mood—it seems it was genuinely filling (or ‘killing’) time.

Our ability to cope with boredom looms large in this story of boredom in the pandemic. Again, emotional self-regulatory ability predicts one’s capacity to cope, and for the boredom prone, the story is not good [19]. Beyond measures of the circumstantial changes induced by the pandemic (i.e., an individual’s extent of physical distancing, engagement in safe outdoor activities, and changes to work status), data showed that difficulty with emotional regulation predicted higher levels of boredom proneness. This was perhaps more starkly evident in a larger sample explored by Bambrah and colleagues [20], who showed that increased feelings of boredom were associated with a preoccupation with the stressors of the pandemic and a difficulty in adapting to those stressors (as measured by the symptoms of adjustment disorder). This relation was exacerbated among the boredom prone. Clearly, a potential target for intervention would be to develop and practice emotional regulation skills to bolster an individual’s capacity to cope with boredom—whether in an unprecedented circumstance or in the more typical environs of daily life.

It has long been known that boredom proneness is associated with a raft of other negative outcomes (see [21] for a review). However, in the context of the pandemic, it becomes important to understand whether such relations—between boredom proneness and drug and alcohol use, for example—are exacerbated. Weiss and colleagues [22] explored this question and found that, indeed, boredom proneness, which itself increased over a number of tested time points, was associated with increased loneliness, elevated use of alcohol and marijuana, and an increase in general distress levels. While both state and trait boredom showed these relationships, it was only trait boredom proneness that continued to show significant relationships with negative outcomes when controlling for the state. In fact, state boredom was associated with a mildly significant *increase* in hope for the future (which is opposite to the relationship observed for boredom proneness and life optimism) [3,11,22]. In fact, the levels of state boredom were even associated with taking *more* steps to avoid contracting COVID-19. As the authors point out, this seemingly counterintuitive finding accords well with functional accounts of boredom [23,24]. That is, if boredom signals the need to engage in something more meaningful or purposeful, then it seems reasonable to assume (without the burden of trait boredom proneness) that the signal comes packaged with some sense of optimism that better engagement is attainable. Herein, too, lies the possibility of another fruitful intervention. If we can reframe what it is that state boredom is telling us—into a positive hope for better engagement—perhaps we can diminish the negative outcomes typically associated with the experience. This is backed up by at least one recent study that showed that beliefs in boredom (i.e., one’s dislike for the state) intensified the experience itself, whereas normalizing boredom was associated with better mental health outcomes [25].

The common thread running through this work is a facet of boredom that, to date, has received little attention—boredom as a threat to our sense and need for agency [26]. In the moment of being bored, we want to be acting effectively in the world but cannot find an outlet for that desire that we imagine will suffice. The constraints of the pandemic laid bare this underappreciated aspect of boredom. In the midst of lockdowns, it became obvious that boredom arose in response to the diminution of agency—so we broke the rules, over-ate, increased the consumption of drugs and alcohol, and generally struggled to regulate our emotional response to the experience. Clearly, this was all more profound for those who, prior to the pandemic, struggled with higher levels of boredom. However, if we can find ways to better regulate our emotions, to recognize that boredom is not to be avoided but to be responded to with adaptive, positive outlets for our desire to act, we might go a long way to living more effectively with the experience, whether in the circumstances similar to those of the past few years or in more typical times.
